# Simultaneous enhancements in photon absorption and charge transport of bismuth vanadate photoanodes for solar water splitting

**DOI:** 10.1038/ncomms9769

**Published:** 2015-10-26

**Authors:** Tae Woo Kim, Yuan Ping, Giulia A. Galli, Kyoung-Shin Choi

**Affiliations:** 1 Department of Chemistry, University of Wisconsin-Madison, Madison, Wisconsin 53706, USA; 2 Department of Chemistry, University of California, Davis, California 95616, USA; 3 Institute for Molecular Engineering, University of Chicago, Chicago, Illinois 60637, USA

## Abstract

n-Type bismuth vanadate has been identified as one of the most promising photoanodes for use in a water-splitting photoelectrochemical cell. The major limitation of BiVO_4_ is its relatively wide bandgap (∼2.5 eV), which fundamentally limits its solar-to-hydrogen conversion efficiency. Here we show that annealing nanoporous bismuth vanadate electrodes at 350 °C under nitrogen flow can result in nitrogen doping and generation of oxygen vacancies. This gentle nitrogen treatment not only effectively reduces the bandgap by ∼0.2 eV but also increases the majority carrier density and mobility, enhancing electron–hole separation. The effect of nitrogen incorporation and oxygen vacancies on the electronic band structure and charge transport of bismuth vanadate are systematically elucidated by *ab initio* calculations. Owing to simultaneous enhancements in photon absorption and charge transport, the applied bias photon-to-current efficiency of nitrogen-treated BiVO_4_ for solar water splitting exceeds 2%, a record for a single oxide photon absorber, to the best of our knowledge.

## Abstract (web summary)

Bismuth vanadate is a promising photoanode for water-splitting, although its performance is limited by its wide bandgap. Here, the authors show that a gentle nitrogen treatment can result in nitrogen doping and oxygen vacancy generation, simultaneously reducing bandgap and increasing charge transport.

## Graphical abstract

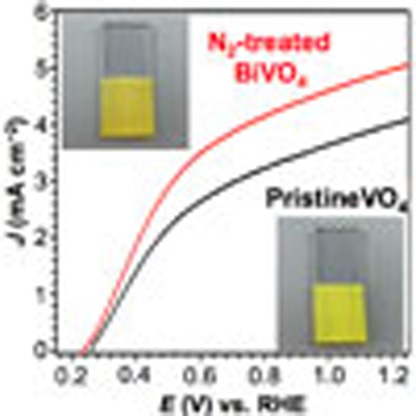

The major challenge for solar hydrogen production is the reduction of hydrogen production cost comparable to that of fossil-based fuels^1^,^2^,^3^. To make solar hydrogen production a reality, a significant cost reduction for the construction of a photoelectrochemical cell (PEC) is critical^1^,^2^,^3^,^4^. Among various semiconductor electrodes (photoelectrodes) for use in PECs, oxide-based photoelectrodes have the possibility of significantly lowering the materials and processing costs while being stable in aqueous media^5^,^6^,^7^. n-Type bismuth vanadate (BiVO_4_) has recently emerged as one of the most promising photoanodes for use in water-splitting PECs^8^,^9^,^10^,^11^. It absorbs a substantial portion of the visible spectrum (bandgap energy, ∼2.5 eV) and has a favourable conduction band edge position, which is very near the thermodynamic H_2_ evolution potential^8^,^9^. As a result, it demonstrated the most negative photocurrent onset potential for water oxidation among all n-type semiconductors (photoanodes) having bandgaps in the visible region. Also, it appears to be fairly stable against chemical and photoelectrochemical corrosion^9^,^10^,^11^. A recent report shows that photoanodes composed of only oxide components, nanoporous BiVO_4_ electrodes coupled with FeOOH and NiOOH as oxygen evolution catalysts^11^, can achieve an applied bias photon-to-current efficiency (ABPE) for water splitting as high as 1.7% in a stable manner (equation for ABPE is shown in Supplementary Information)^12^. This result demonstrated the possibility for constructing a PEC for water splitting using only inexpensive and easy-to-process oxide components.

One of the major limitations of BiVO_4_ for further improving the solar energy conversion efficiency is its bandgap (∼2.5 eV), which fundamentally limits photon absorption. Considering that there are a substantial number of photons in the 2.0–2.5 eV region of the solar spectrum, a reduction in bandgap by even 0.1–0.3 eV can result in a significant efficiency increase^13^,^14^. For example, if a bandgap is reduced from 2.5 to 2.3 eV, the maximum photocurrent (*J*_max_) will increase from 6.47 to 9.12 mA cm^–2^ assuming 100% incident photon-to-current conversion efficiency (IPCE) for the photons that enable bandgap transition^13^. However, most previous doping studies of BiVO_4_ were to increase carrier densities^15^,^16^,^17^,^18^. Studies reporting the change in photon absorption of BiVO_4_ have been scarce and no clear elucidation of the effect of the dopants on the electronic band structures has been reported^19^,^20^. Considering that the valence band maximum (VBM) of BiVO_4_ is significantly more positive than water oxidation potential, but the conduction band minimum (CBM) of BiVO_4_ is very close to the reduction potential of water^8^,^9^,^21^, the reduction in bandgap of BiVO_4_ will be the most advantageous if it is achieved by raising the VBM.

In this study, we report that mild annealing treatment of nanoporous BiVO_4_ under N_2_ flow results in nitrogen incorporation into the oxygen sites, which effectively decreases the bandgap while also improving carrier mobility. In addition, we discover that the same N_2_ treatment generates oxygen vacancies that can increase the majority carrier density. The effect of nitrogen incorporation and oxygen vacancies on the electronic band structure and charge transport of BiVO_4_ are systematically elucidated by *ab initio* calculations, which corroborate well with the experimental results. Owing to concurrent enhancement in photon absorption, carrier density and carrier mobility, the N_2_-treated BiVO_4_ electrode, when paired with oxygen evolution catalysts, breaks the wall of 2% ABPE for solar water splitting, a first for a single oxide photoelectrode.

## Results

### Synthesis and characterization

Nanoporous BiVO_4_ electrodes were first prepared by the method described in a recent study, which involves electrochemical deposition of BiOI followed by thermal and chemical conversion to BiVO_4_ (ref. 11). Nitrogen doping of BiVO_4_ was achieved by annealing the BiVO_4_ electrodes at 350 °C for 2 h while flowing N_2_. This is an exceptionally gentle procedure for nitrogen doping considering that nitridation or nitrogen doping of oxides is generally achieved by annealing with a flow of NH_3_ at much higher temperatures (≥500 °C)^22^,^23^,^24^,^25^. Annealing under N_2_ flow may create oxygen deficiencies in oxide compounds but is usually considered insufficient for nitrogen doping. We believe that the nanoparticulate nature of the BiVO_4_ electrodes played a role in enabling nitrogen doping under such mild conditions.

**Figure 1:**
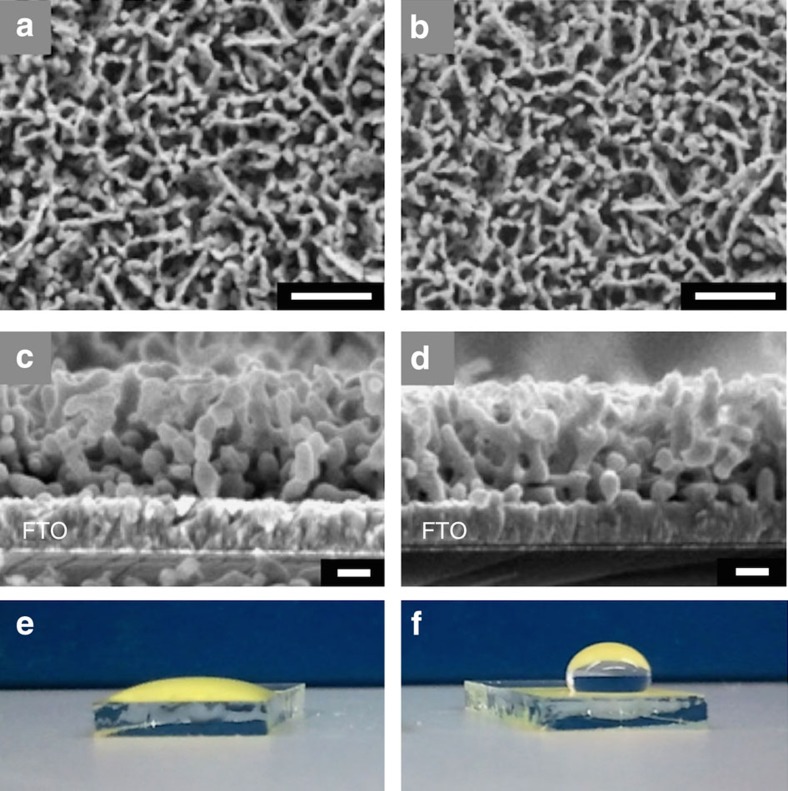
SEM images and photographs. Top-view and side-view SEM images of (**a**,**c**) BiVO_4_ and (**b**,**d**) N_2_-treated BiVO_4_. Photographs of a water droplet placed on (**e**) BiVO_4_ electrode and (**f**) N_2_-treated BiVO_4_ electrode. Scale bars, 1 μm for (**a**,**b**) and 200 nm for (**c**,**d**).

The scanning electron microscopy images and X-ray diffraction studies of N_2_-treated BiVO_4_ electrodes do not show noticeable changes from those of untreated BiVO_4_ (Fig. 1a–d; Supplementary Fig. 1). The first indication of nitridation was observed when a drop of water was placed on the surface of these electrodes to compare their hydrophilicities (Fig. 1e,f). While the BiVO_4_ sample showed a high affinity to water, as any oxide surface would, the N_2_-treated sample showed strong hydrophobicity, suggesting the possibility of nitridation, which can considerably change the hydrophilicity of the surface.

**Figure 2:**
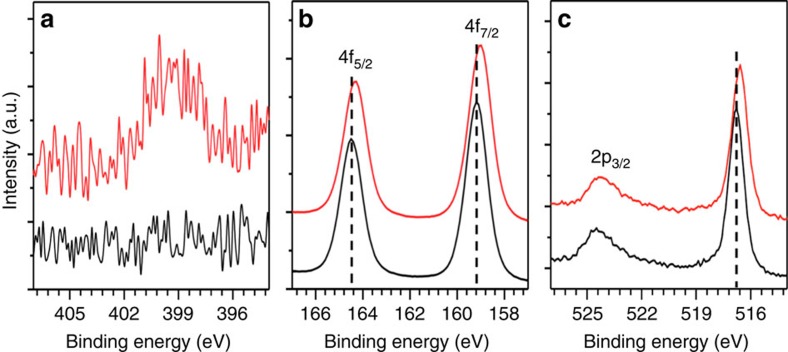
XPS of BiVO_4_ and N_2_-treated BiVO_4_. (**a**) 1*s* peaks of N, (**b**) 4*f* peaks of Bi and (**c**) 2*p* peaks of V for BiVO_4_ (black) and N_2_-treated BiVO_4_ (red).

The incorporation of nitrogen into the BiVO_4_ lattice was confirmed by electron probe microanalyzer (EPMA) and X-ray photoelectron spectroscopy (XPS). For charge-balanced incorporation of nitrogen, three O^2−^ ions need to be removed for every two N^3−^ ions, creating one oxygen vacancy. Therefore, the formula of nitrogen-doped BiVO_4_ can be written as BiVO_4−1.5*x*_N_*x*_ if no other types of oxygen vacancies are present. The EPMA result showed that *x* is 0.31±0.05 (that is, BiVO_3.54_N_0.31_). XPS study also confirmed the presence of nitrogen in the BiVO_4_ lattice by showing a N 1*s* peak (Fig. 2a) with the nitrogen content, *x*, estimated to be 0.34±0.02, which is comparable to the value obtained by EPMA. The location of the N 1*s* peak (397–402 eV) agrees well with that of nitrogen incorporated into the oxide lattice^25^,^26^. The incorporation of N into the BiVO_4_ lattice was further confirmed by Raman spectra that show significant broadening of various V–O stretching and VO_4_^3−^ deformation modes of the bulk sample (Supplementary Fig. 2)^27^,^28^. This indicates that unlike other oxides, nitrogen incorporation into BiVO_4_ can occur using N_2_ as the N source at mild temperatures. In fact, there exist two previous studies reporting nitrogen incorporation into vanadium oxides by annealing under N_2_ flow^29^,^30^, suggesting relatively easy nitrogen incorporation into vanadium-based oxides.

The XPS study also revealed that the Bi 4*f* and V 2*p* peaks of N_2_-treated BiVO_4_ were shifted to the lower binding energy (Fig. 2b,c). This should be due to the changes in local coordination environments of Bi and V ions, including a change in ligand type (O to N) and a decrease in coordination number due to oxygen vacancies compensating the charge difference between O^2−^ and N^3−^. In addition, the possibility that the N_2_ treatment created additional oxygen vacancies^31^,^32^, which would result in partial reduction of Bi^3+^ and V^5+^ ions, cannot be excluded.

**Figure 3:**
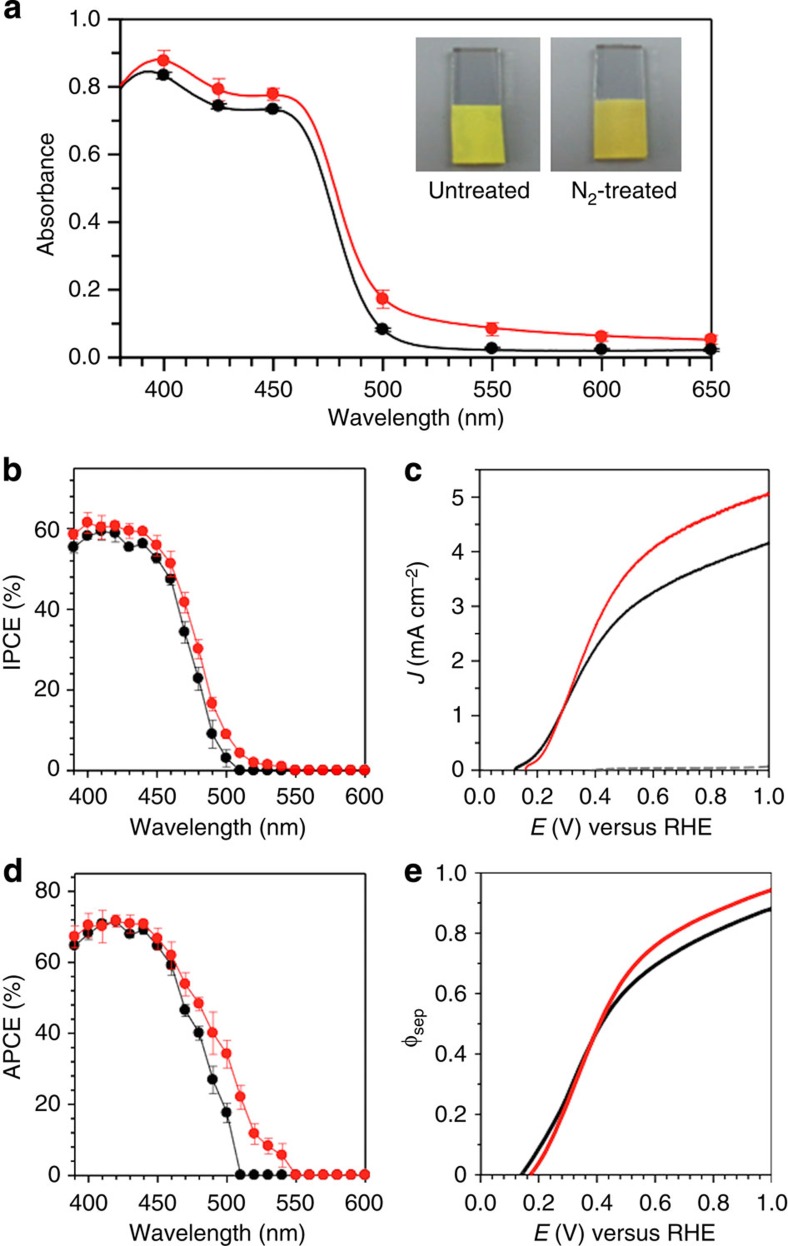
Optical and photoelectrochemical properties. (**a**) Ultraviolet–visible absorption spectra with photographs of samples, (**b**) IPCE at 0.6 V versus RHE, (**c**) *J*–*V* plots for sulfite oxidation under AM 1.5G, 100 mW cm^–2^ illumination (scan rate, 10 mV s^–1^), (**d**) APCE at 0.6 V versus RHE and (**e**) *φ*_sep_s calculated from the *J*–*V* plots for BiVO_4_ (black) and N_2_-treated BiVO_4_ (red). A 0.5 M phosphate buffer (pH 7.2) containing 1 M Na_2_SO_3_ was used as the electrolyte. The error bars were obtained by taking the s.d. values of measurements on three different samples.

The ultraviolet–visible spectra of BiVO_4_ and N_2_-treated BiVO_4_ electrodes are shown in Fig. 3a. The N_2_-treated sample clearly showed a shift of the bandgap absorption to lower energy compared with untreated BiVO_4_. This agrees well with the darker yellow colour of the N_2_-treated BiVO_4_ (Fig. 3a, inset). The N_2_-treated BiVO_4_ also shows a significant absorption before the absorption edge (wavelength >550 nm). This is most likely due to N_2_ treatment generating significant disorder in the atomic arrangement (for example, O defects) on the surface of the high surface area nanoporous BiVO_4_ electrode while incorporating nitrogen, creating interband states in the bandgap region^33^,^34^. The absorption due to these interband states makes it difficult to accurately assess the onset for the bandgap transition.

### Photo-oxidation of sulfite

To determine the onset of photon absorption that contributes to the photocurrent generation, wavelength-dependent photocurrent was measured using monochromatic light for the photo-oxidation of sulfite and the IPCE was calculated. Since the oxidation kinetics of sulfite is fast, surface electron–hole recombination is negligible during photo-oxidation of sulfite. Therefore, photo-oxidation of sulfite enables a more precise estimation of photocurrent onset compared with photo-oxidation of water which typically suffers from slow oxidation kinetics and surface recombination.

The IPCE result obtained at 0.6 V versus reversible hydrogen electrode (RHE) shows that the photocurrent onset of the N_2_-treated sample lies between 540 and 550 nm (∼2.27 eV) while that of the untreated sample lies between 500 and 510 nm (∼2.46 eV), which indicates a bandgap reduction of ∼0.2 eV for the N_2_-treated sample (Fig. 3b). The *J*–*V* plots of BiVO_4_ and N_2_-treated BiVO_4_ obtained using AM 1.5G illumination for sulfite oxidation are also shown in Fig. 3c. The N_2_-treated BiVO_4_ electrode shows a significantly higher photocurrent than the BiVO_4_ electrode. For example, the N_2_-treated sample generated a photocurrent density of 4.16±0.41 mA cm^–2^ at 0.6 V versus RHE while BiVO_4_ generated 3.27±0.31 mA cm^–2^ at the same potential, corresponding to a 27% increase. This is the first example showing the generation of photocurrent higher than 4 mA cm^–2^ for any photo-oxidation reaction by any photoanode reported to date at a potential as low as 0.6 V versus RHE.

If the photocurrent enhancement is due only to the enhanced photon absorption by the bandgap reduction, the N_2_-treated sample should show an increase in IPCE but not in the absorbed photon-to-current conversion efficiency (APCE). However, the N_2_-treated sample showed a considerable enhancement in APCE, particularly between 470 and 550 nm (Fig. 3d), indicating that the N_2_ treatment also improved electron–hole separation. Indeed, the electron–hole separation yield, *φ*_sep_, of the N_2_-treated sample, which was calculated from the *J*–*V* plots obtained for sulfite oxidation, shows about an 8–10% increase at *E*>0.45 V versus RHE (Fig. 3e). For example, the electron–hole separation yields at 0.6 V versus RHE are 0.70±0.04 and 0.76±0.07 for untreated and N_2_-treated BiVO_4_, respectively. This means that the 27% photocurrent enhancement observed at 0.6 V for the N_2_-treated sample is partially due to an increase in photon absorption and partially due to an increase in electron–hole separation (see the Methods section and Supplementary Table 1 for detailed calculations).

One plausible mechanism for N_2_ treatment to increase *φ*_sep_ is to increase the carrier density by creating extra oxygen vacancies, which can serve as donors, in addition to the oxygen vacancies created for charge-balanced substitution of N^3−^ with O^2−^. This can result in an improvement in electron transport and, therefore, in electron–hole separation. The carrier densities in BiVO_4_ and N_2_-treated BiVO_4_ electrodes were compared by obtaining Mott–Schottky plots (Supplementary Fig. 3). For nanoporous films, since the regions that contribute to space-charge layer capacitance and the corresponding surface area cannot be precisely quantified, accurate quantitative analysis of carrier density using Mott–Schottky analysis is not possible. However, since the samples compared in this study have exactly the same morphologies (particle sizes, aggregations and connectivities), relative qualitative comparison of carrier densities should be possible by comparing the magnitude of the slopes. The result shows that the slope of N_2_-treated BiVO_4_ is ∼1/7 of the slope of BiVO_4_, indicating that the N_2_-treated BiVO_4_ electrodes possess extra oxygen vacancies that serve as electron donors. This suggests that the record-high photocurrent shown in Fig. 3c is due to improved electron transport properties as well as bandgap reduction.

An increase in n-type carrier density should result in a shift of flatband potential (*E*_FB_) to the negative direction. However, both the photocurrent onset and the *E*_FB_ of the N_2_-treated sample are more positive than those of BiVO_4_ (Fig. 3c; Supplementary Fig. 3). This is because the Helmholtz layer potential drop (*V*_H_) of the N_2_-treated BiVO_4_ electrode, which is another factor that affects the position of *E*_FB_ (ref. 35), is more positive than that of BiVO_4_, which is indicated by zeta potential measurements (Supplementary Fig. 4). The change in *V*_H_ of the N_2_-treated sample appears to cancel out the effect of an increased donor density on *E*_FB_.

### Density functional theory calculations

The effect of charge-balanced N incorporation (that is, replacing three O^2−^ with two N^3−^) and of oxygen vacancies on the electronic band structure of BiVO_4_ were studied using density functional theory (DFT) calculations, to gain insights into the changes occurring in BiVO_4_ upon nitridation. Our experimental results show that in the N_2_-treated BiVO_4_, both N substitution and O vacancies are present at the same time, yielding a complex, combined effect on the electronic structure of BiVO_4_. Before investigating such combined effect, we discuss the electronic properties of N substitution and oxygen vacancies separately.

We first performed spin-polarized calculations without addressing the charge imbalance between O^2−^ and N^3−^. We used the Perdew–Burke–Ernzerh (PBE)^36^ exchange correlation functional for pure BiVO_4_ and BiVO_4_ with 12.5% neutral O replaced with neutral N (1:1 substitution). We found that in the presence of N the bandgap of BiVO_4_ was reduced by 0.5 eV (Supplementary Fig. 5), mainly because of the hybridization of N and O 2*p* states close to VBM. The presence of extra holes due to N substitution leads to two localized empty bands (level labelled ‘a' in Supplementary Fig. 5b) inside the bandgap of BiVO_4_. These bands will no longer be present when N substitution accompanies charge-balancing O vacancies, which will serve as electron donors and compensate for excess holes from N substitution, as discussed below (see also Supplementary Fig. 6 for the effect of artificially adding an electron per nitrogen to compensate for the excess holes from N substitution).

To understand the effect of O vacancies separately, we performed spin-polarized calculations at two different vacancy concentrations of 6 and 1.5%, using PBE and PBE with Hubbard *U*-corrections^37^ (PBE+*U*, effective *U*(V)=2.7 eV)^17^, to verify the robustness of our results with respect to the functional adopted in our calculations. Both at the PBE and PBE+*U* levels of theory, at the two concentrations, we found that the presence of O vacancies, which were created by removing neutral O atoms, did not lead to any modification of the bandgap of BiVO_4_ (for details see Supplementary Figs 7 and 8). We also observed the formation of localized, filled states inside the bandgap of pristine BiVO_4_. Interestingly, the spin density of the gap states is localized on V, as shown in Supplementary Fig. 8, causing local lattice distortions (in addition to those induced by missing O atoms), and indicating the formation of small polarons. Indeed, small polarons are likely to form in oxides such as BiVO_4_ that exhibit strong electron–phonon interaction, as indicated, for example, by the large difference between the high frequency and static dielectric constants *ɛ* (the high frequency and static *ɛ*, averaged over the three lattice directions, are 6.9 and 52, respectively, as computed by density functional perturbation theory^38^ for pure BiVO_4_).

Our results on the formation of small polarons are consistent with recent mobility measurements on single crystal *n*-type BiVO_4_, which reported very low electron mobilities^39^ (∼0.2 cm^–2^ V^–2^ s^–1^) and suggested that the conduction of electrons is dominated by small polaron hopping. Previous calculations showed that O vacancies lead to shallow donor levels in BiVO_4_ (ref. 31), with negative ionization energies for *E*(V^0^/V^2+^) and that the only stable charged state for an O vacancy in BiVO_4_ is *q*=+2 due to the spontaneous ionization of two electrons, regardless of the Fermi level position^40^. These results are consistent with our finding that the two electrons present at an O vacancy site are ionized and localized at V atoms, thus forming small polarons. Similar findings for SrTiO_3_ have also been discussed in a recent study by Janotti *et al*.^41^ We also computed the small polaron binding energy^42^, that is, the energy difference between bound small polarons and free carriers. We found a non-negligible value of ∼0.50 eV indicating that the formation of small polarons is energetically favourable (see Methods for calculation details).

**Figure 4:**
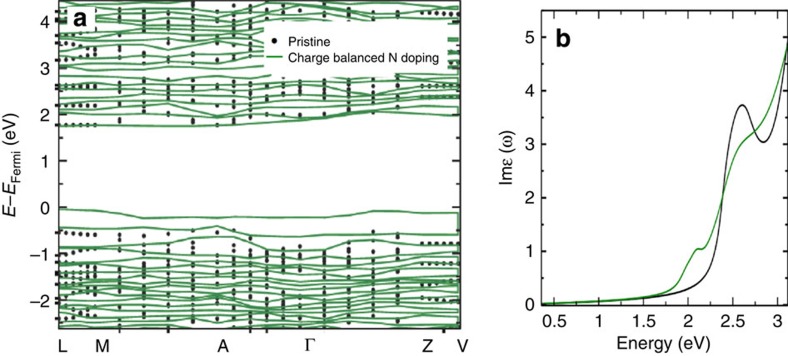
Comparison of pristine and charge-balanced N-doped BiVO_4_. (**a**) Band structures and (**b**) simulated absorption spectra (imaginary part of dielectric function) of pristine BiVO_4_ (black) and BiVO_4_ with charge-balanced N-doping (9% O replaced with 6% N and 3% O vacancy) (green). An artificial broadening 0.08 eV is applied to take into account the finite temperature and phonon effects. The highest occupied level at 0 K of BiVO_4_ with charge-balanced N-doping was taken as the Fermi level (*E*_Fermi_).

Having established the separate effects of N substitution and O vacancies on the electronic structure of BiVO_4_, we now turn to analyze their combined effect. In the case of charge-balanced N substitution (9% O replaced with 6% N and 3% O vacancy) we found that no states are formed in the bandgap of BiVO_4_, as shown in Fig. 4a. This is because the electrons ionized from O vacancies compensate for the holes generated by N substitution, thus balancing the charge. No small polaron is formed at the vanadium sites in this case. The important finding is that the bandgap is still lowered by 0.3–0.4 eV (depending on the location in the lattice of the substituted N) compared to that of pure BiVO_4_. The computed projected density of states (as shown in Supplementary Fig. 9) showed that the VBM has contributions from both O and N 2p states, similar to the case of N substitution discussed earlier. With lower doping concentration (4.5% O replaced with 3% N and 1.5% O vacancy), the VBM moves upward by 0.2 eV compared with that of pure BiVO_4_. In sum, we found that in the presence of N substitution and charge-compensating O vacancies, the bandgap decrease is similar to that obtained in the case where only substitutional N is present, and the bandgap decrease is more pronounced with increasing N concentration.

The information on energy levels provided by band structure calculations is insufficient to investigate light absorption efficiency of BiVO_4_. Hence, we also computed the optical transition probabilities from valence to conduction states for pristine and charge-balanced N-doped BiVO_4_ (9% O replaced with 6% N and 3% O vacancy) and simulated absorption spectra using the random phase approximation including local field effects^43^. Although this approximation neglects excitonic effects, we expect it to be accurate to account for changes in absorption spectra when N is added to the sample^44^. We estimated the change of the exciton binding energy before and after doping by using a hydrogen-like model and we found it is of the order of 0.05 eV (see Methods for details). The spectra were averaged over three lattice directions to compare with experimental results obtained from polycrystalline samples. The resulting simulated absorption spectra show that the absorption edge of N-doped BiVO_4_ is lower than that of the pristine BiVO_4_ by about 0.3 eV (Fig. 4b), confirming that charge-balanced N substitution can indeed enhance visible light absorption at lower energy. This result agrees well with our experimental observation that incorporating ∼8% N results in a 0.2 eV reduction of the bandgap.

**Figure 5:**
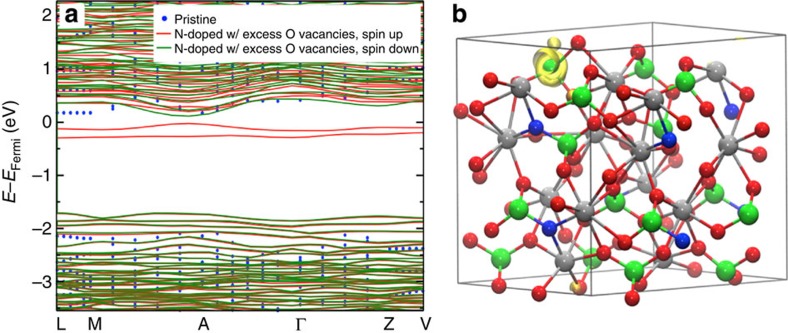
Calculation results for N-doped BiVO_4_ with excess O vacancies. (**a**) Comparison of band structures of pristine BiVO_4_ (blue dot) and N-doped BiVO_4_ with excess O vacancies (6% N and 6% O vacancy) with spin up (red) and spin down (green) configurations. The highest occupied level at 0 K of N-doped BiVO_4_ with excess O vacancies (spin up configuration) was taken as the Fermi level (*E*_Fermi_). (**b**) Spin density map of N-doped BiVO_4_ with excess O vacancies showing the localization of an electron on V. The spin density isosurface (0.0049, e bohr^−3^) of an isolated band (around the Fermi level at the *Γ* point) is shown in yellow; atoms are represented by spheres: V (green), Bi (silver), O (red) and N (blue). Projected density of states is shown in Supplementary Fig. 10.

Our experimental results suggest that in addition to the O vacancies caused by charge-balanced replacement of O^2−^ with N^3−^, additional oxygen vacancies are present serving as electron donors in N_2_-treated BiVO_4_. Therefore, we performed calculations of N-doped BiVO_4_ with excess O vacancies (6% N and 6% O vacancy) (Fig. 5; Supplementary Fig. 10), where half of the O vacancies balance the charges of substitutional N atoms, and the other half provide extra electrons to form polarons. We observed both a reduction of the bandgap by 0.3 eV (Fig. 5a), compared with that of pristine BiVO_4_, and the formation of small polarons localized on V atoms (Fig. 5b).

We also note that N-doping increases the mobility of small polarons by changing the static dielectric constant of BiVO_4_ (ref. 45). Our computation shows that small polarons' activation energy of N-doped BiVO_4_ (6% N substitution and 3% O vacancies) is lowered by 1.1% and, as a result, the electron mobility is increased by 25% according to the small polaron model (see the Methods section for the computation details)^39^,^42^. This means that the measured, enhanced *φ*_sep_ of the N_2_-treated sample is not only due to an increase in carrier density but also due to an increase in carrier mobility. To confirm this, we prepared H_2_-treated BiVO_4_ that has the same O vacancy concentration and, therefore, the same carrier density as the N_2_-treated sample but no N incorporation (Supplementary Fig. 11)^31^. By comparing electron transport properties of the two samples using APCEs and photocurrents generated by front-side and back-side illuminations (Supplementary Figs 11 and 12)^16^,^46^,^47^,^48^, we observed that the electron transport of N_2_-treated samples is more enhanced than that of H_2_-treated samples. This result suggests that other than the carrier density increase by O vacancies that are common for H_2_-treated and N_2_-treated samples, the N_2_-treated samples have an additional factor improving electron transport. This agrees well with the result from the polaron activation energy calculation that N incorporation results in an increase in polaron mobility.

Altogether, our experimental and computational results show that the simple and mild N_2_ treatment used in this study simultaneously enhanced photon absorption, carrier density and carrier mobility, all of which are critical for improving the photoelectrochemical properties of BiVO_4_.

### Photo-oxidation of water

The ability of N_2_-treated BiVO_4_ electrode to photoelectrolyze water was examined in a 0.5 M phosphate buffer (pH 7.2) after oxygen evolution catalysts (OECs), FeOOH and NiOOH, were photodeposited on the N_2_-treated BiVO_4_ surface sequentially; a FeOOH layer was first photodeposited on the N_2_-treated BiVO_4_ surface followed by the deposition of a NiOOH (see Methods for the synthesis details). It was reported that the BiVO_4_/FeOOH interface minimizes electron–hole recombination at the BiVO_4_/FeOOH junction and the NiOOH/electrolyte surface makes the surface charge more favourable while concurrently improving the water oxidation kinetics^11^. As a result, the dual catalyst layer structure provided the best performance compared with single layer or the OEC layers with the reversed order.

Since the surface of the N_2_-treated BiVO_4_ electrode is expected to be quite different from that of the untreated BiVO_4_ electrode (for example, surface charge and surface termination), the N_2_-treated BiVO_4_/FeOOH interface may no longer be ideal. However, depositing the same OECs on BiVO_4_ and N_2_-treated BiVO_4_ would allow us to examine any changes at the BiVO_4_/OEC junction and for solar water oxidation caused solely by N_2_ treatment.

**Figure 6:**
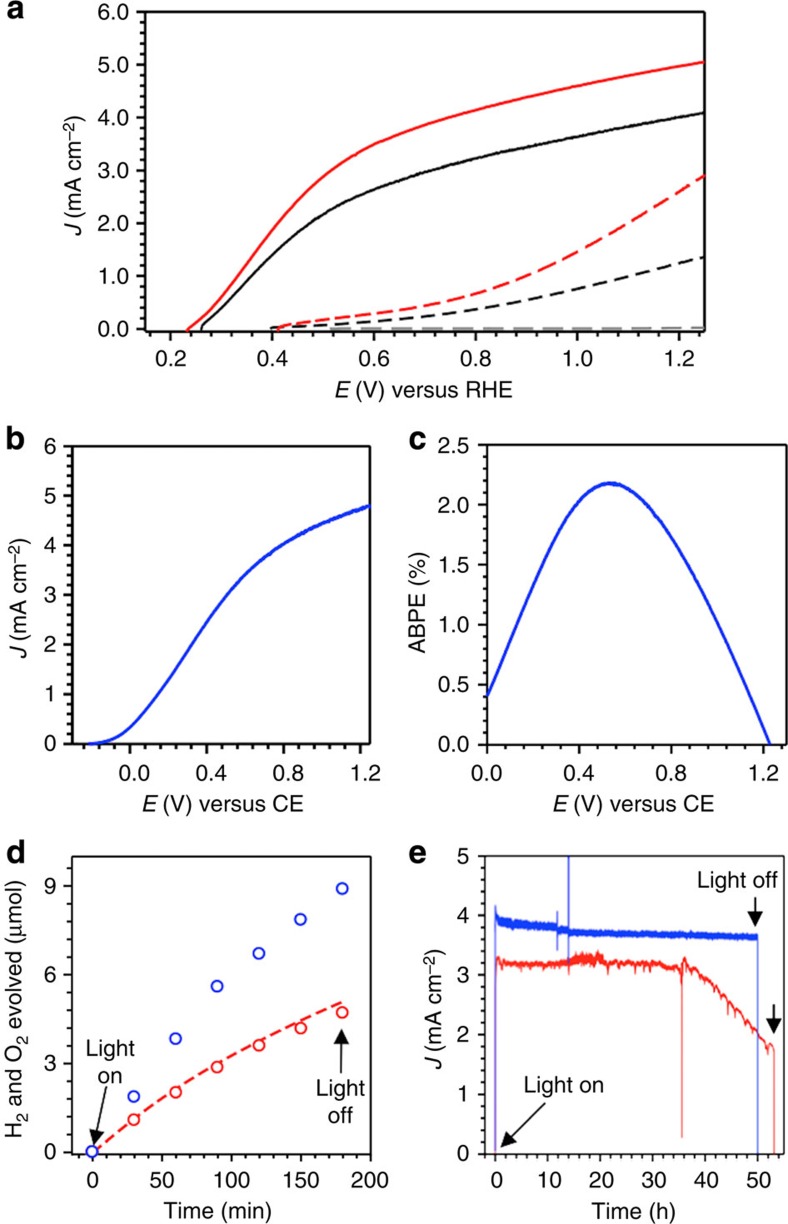
Photoelectrochemical water-splitting performance. (**a**) *J*–*V* plots of BiVO_4_/FeOOH/NiOOH (black, solid), N_2_-treated BiVO_4_/FeOOH/NiOOH (red, solid), BiVO_4_ (black, dashed), and N_2_-treated BiVO_4_ (red, dashed) measured in a 0.5 M phosphate buffer (pH 7.2) under AM 1.5G illumination using a three-electrode cell. Grey dashed lines show dark current of N_2_-treated BiVO_4_/FeOOH/NiOOH. (**b**) *J–V* plot and (**c**) ABPE of N_2_-treated BiVO_4_/FeOOH/NiOOH obtained using a two-electrode cell for solar water splitting (CE: counter electrode). (**d**) Detection of H_2_ (blue) and O_2_ (red) produced by N_2_-treated BiVO_4_/FeOOH/NiOOH at 0.6 V versus counter electrode. The red dashed line represents the amount of O_2_ calculated assuming 100% Faradaic efficiency. (**e**) *J–t* plots of N_2_-treated BiVO_4_ for sulfite oxidation (blue) and N_2_-treated BiVO_4_/FeOOH/NiOOH for water oxidation (red) at 0.6 V versus RHE.

The results show that N_2_ treatment increased the photocurrent density for water oxidation at 0.6 V versus RHE from 2.71 to 3.47 mA cm^–2^ (Fig. 6a). The *J*–*V* curve was also measured using a two-electrode cell to calculate ABPE (Fig. 6b). The highest ABPE, 2.2%, was achieved at 0.58 V, which is the record-high ABPE attained by a single oxide photon absorber to date (Fig. 6c). The Faradaic efficiency for O_2_ evolution at 0.6 V versus RHE was 96% (Fig. 6d). The ratio of H_2_:O_2_ produced was 1.81:1 (Fig. 6d). Considering that there are no competing reactions available at the cathode, the slight deviation from the stoichiometric H_2_/O_2_ production is due to the leak of H_2_ during manual sampling of H_2_ for gas chromatography analysis.

The long-term stability of N_2_-treated BiVO_4/_FeOOH/NiOOH for water oxidation was examined by measuring *J–t* plots at 0.6 V versus RHE and was compared with the long-term stability of N_2_-treated BiVO_4_ for sulfite oxidation at the same potential (Fig. 6e). The photocurrent for sulfite oxidation was stable for 50 h and no sign of decay was observed, suggesting that the presence of nitrogen and oxygen vacancies in the lattice are stable under illumination and electrical bias. The photocurrent for water oxidation was stable for 30 h but after 30 h it gradually decreased. Considering the photostability of N_2_-treated BiVO_4_ for sulfite oxidation and the previously reported photostability of undoped BiVO_4_ combined with the same OECs for water oxidation^11^, the instability observed during photo-oxidation of water is most likely due to the instability of the N_2_-treated BiVO_4_/OEC junction resulting in the loss of OECs over time, which was confirmed by XPS studies (Supplementary Fig. 13). These results suggest that while N_2_-treated BiVO_4_ itself is compositionally stable, it will require new OECs to form a more stable N_2_-treated BiVO_4_/OEC interface to achieve a long-term stability for photo-oxidation of water.

## Discussion

Annealing of nanoporous BiVO_4_ at 350 °C under N_2_ flow resulted in substitutional incorporation of nitrogen and the formation of O vacancies, which simultaneously enhanced photon absorption, carrier density and carrier mobility. The effect of N incorporation and O vacancies in the BiVO_4_ lattice were thoroughly examined experimentally and computationally to provide new understanding of the electronic band structures and photoelectrochemical properties of BiVO_4_. Charge-balanced N-doping (that is, 9% O replaced with 6% N and 3% O vacancy) reduced the bandgap of BiVO_4_ by elevating the VBM without introducing mid-gap states in the bandgap region. N-doping also decreased the static dielectric constant of BiVO_4_ and improved the mobility of electrons, which are small polarons. In addition to the O vacancies required for charge-balanced N-doping, the N_2_-treated BiVO_4_ contained additional oxygen vacancies, which served as electron donors in N_2_-treated BiVO_4_. As a result, the major carrier density as well as the major carrier mobility of BiVO_4_ could be increased by the simple N_2_ treatment. The experimental and theoretical studies reported herein may serve as guidelines to further improve photon absorption and charge transport of BiVO_4_-based photoanodes for more practical and efficient solar water splitting. For example, predicting and incorporating a dopant that can simultaneously improve photon absorption and charge transport may serve as an effective strategy to improve photoelectrochemical performance. Future studies should also include identifying OECs that can optimally interface with BiVO_4_ electrodes with modified compositions.

## Methods

### Nitrogen incorporation into BiVO_4_

The nanoporous BiVO_4_ electrode used in this study was prepared using the same method reported in our recent paper^11^,^49^. Nitrogen incorporation was achieved by annealing the BiVO_4_ electrodes at 350 °C for 2 h in a tube furnace while flowing N_2_. This annealing condition was identified as optimal to generate samples with the best photoelectrochemical properties after trying various annealing temperatures (300–450 °C, ramping rate=5 °C min^–1^) and annealing times (1–3 h). The N incorporation shown in this study is the maximum N incorporation that can be achieved by N_2_ treatment. Increasing temperature or duration of N_2_ treatment did not increase the N incorporation. When the duration of N_2_ treatment is shortened, the amount of N incorporated could be reduced but the photocurrent enhancement was also reduced. For example, when the annealing time at 350 °C while flowing N_2_ was reduced from 2 h to 30 min, the N content, *x*, in BiVO_4−1.5*x*_N_*x*_ was reduced from=0.33 to 0.10 judging from the XPS results and the photocurrent density at 0.6 V versus RHE was reduced from 4.2 to 3.4 mA cm^–2^.

### Photodeposition of FeOOH/NiOOH OECs on N_2_-treated BiVO_4_ electrodes

The photodeposition of dual-layer oxygen evolution reaction catalysts (that is, FeOOH and NiOOH) on the surface of the N_2_-treated BiVO_4_ photoanode were performed using the method described in our recent paper^11^ with a few modifications. Before photodeposition, the N_2_-treated BiVO_4_ was first immersed in 1 M NaOH solution for ∼1–2 min to ensure the wetting of its hydrophobic surface. After rinsing the surface with DI water, the photodeposition of FeOOH was carried out in a 0.1 M FeSO_4_ solution (pH 3.2–3.8) while gently stirring. Before photodeposition, the solution was purged with nitrogen gas for 1 h. An undivided three-electrode cell composed of a N_2_-treated BiVO_4_ working electrode, a Pt counter electrode, and a Ag/AgCl (4 M KCl) reference electrode, was used. A 300 W Xe arc lamp (Oriel Newport) with an AM 1.5G filter, neutral density filters and a water filter (IR filter) was used as the light source. The light was illuminated through the FTO contact (back-side illumination) and the light intensity at the FTO surface was adjusted to be 1 mW cm^–2^ using a research radiometer (International Light, IL 1700) coupled with a thermopile detector (International Light, SED623/H/N/K15). To facilitate photodeposition, an external bias of ca. 0.26–0.31 V versus Ag/AgCl (4 M KCl), which was the open circuit potential of the N_2_-treated BiVO_4_ electrodes in the solution in dark, was applied using a potentiostat (BioLogic SP-200). After the photodeposition of FeOOH, the electrodes were rinsed with DI water and dried at room temperature. Among various photodeposition times tested (that is, 10–60 min), deposition for *ca.* 45 min (passing a total charge of 65–70 mC cm^–2^) was identified to be optimum to maximize photocurrent generation for water oxidation.

Photodeposition of NiOOH was carried out on the N_2_-treated BiVO_4_/FeOOH electrodes using a 0.1 M NiSO_4_ solution with pH adjusted to 6.3–7.0 by adding NaOH. To facilitate photodeposition, an external bias of ca. 0.12–0.16 V versus Ag/AgCl (4 M KCl), which was the open circuit potential of the N_2_-treated BiVO_4_/FeOOH electrodes in the solution in dark, was applied. The optimum deposition time was ca. 25 min (passing a total charge of 20–22 mC cm^–2^). After photodeposition of NiOOH, NiOOH was also electrochemically deposited by applying +1.2 V versus Ag/AgCl (4 M KCl) for 1 min. This was to deposit NiOOH on any bare BiVO_4_ or FTO surfaces exposed to the electrolyte. After deposition, the electrodes were washed with DI water and dried at room temperature. The molar ratio of Fe/Ni in optimized N_2_-treated BiVO_4_/FeOOH/NiOOH electrodes was estimated to be 4.92±1.87 by energy-dispersive spectroscopy (using three samples at the 95% confidence level).

### Characterization

The purity and crystal structure of the synthesized electrodes, pristine BiVO_4_ and N_2_-treated BiVO_4_, were examined by X-ray diffraction (Bruker D8 Advanced PXRD, *λ*=1.5418 Å, 298 K, Ni-filtered Cu K_α_-radiation). The crystal morphologies of the electrodes were examined with scanning electron microscopy using a LEO 1530 microscope at an accelerating voltage of 5 kV. The chemical composition of Bi/V in the BiVO_4_ electrodes was confirmed to be 0.98±0.02 by a Hitachi S3400-N microscope equipped with an energy-dispersive X-ray spectrometer (Thermo Fisher Scientific Inc.). For the quantitative analysis of the amount of nitrogen doping in N_2_-treated BiVO_4_, EPMA employing a CAMECA SX-50 apparatus was conducted. In sample preparation, the anode material on the FTO film was scraped off and placed on a carbon tape. A precisely quantitative analysis of nitrogen was achieved by calibration using a high-purity GaN as a standard reference. Ultraviolet–visible absorption spectra were obtained on a Cary 5000 Ultraviolet–visible spectrophotometer (Agilent), in which the sample electrode was fixed in the centre of an integrating sphere to measure all light reflected and transmitted to accurately assess the absorbance. FTO glass was used as the reference for these absorption measurements. XPS spectra were recorded using a K-Alpha X-ray photoelectron spectrometer (Thermo Scientific Inc.) equipped with a monochromated Al K_α_ line as the X-ray source. The binding energies were calibrated with respect to the residual C (1*s*) peak at 284.6 eV. Raman spectra of the pristine BiVO_4_ and the N_2_-treated BiVO_4_ electrodes were recorded at room temperature using a Raman spectrometer (Thermo Scientific DXR smart Raman spectrometer). A red laser (780 nm) was used as excitation source. The electrodes were placed such that the BiVO_4_ and the N_2_-treated BiVO_4_ side of electrodes faced the laser source. Capacitance measurements for Mott–Schottky plots were obtained using a SP-200 potentiostat/EIS (BioLogic Science Instrument) with an EC-Lab software package. A sinusoidal modulation of 10 mV was applied at frequencies of 0.5 and 1 kHz. The three-electrode setup was used in a 0.5 M phosphate buffer solution (pH 7.2). All electrodes were masked with epoxy resin to expose the same geometrical area (ca. 0.1 cm^2^) for this measurement. The zeta potential measurements of pristine BiVO_4_ and N_2_-treated BiVO_4_ were conducted using a zeta potential analyser (Micromeritics NanoPlus-2), which measures electrophoretic mobilities of charged particles to calculate zeta potentials. This was to compare Helmholtz layer potential drop (*V*_H_) of the pristine and N_2_-treated BiVO_4_ electrodes (equation 1), which is another factor that affects the position of flatband potential (*E*_FB_) (equation 2)^35^.


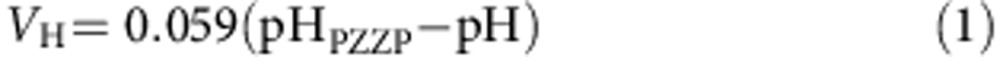



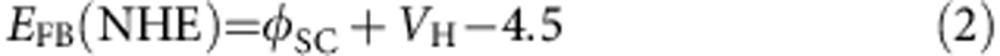


The point of zero zeta potential (pH_PZZP_) of BiVO_4_ and N_2_-treated BiVO_4_ could not be directly measured because BiVO_4_ is not chemically stable near its pH_PZZP._ However, zeta potentials at pH 7.2 solution could still be used to qualitatively compare the pH_PZZP_s of BiVO_4_ and N_2_-treated BiVO_4_ and their effects on E_FB_. For the zeta potential measurement, BiVO_4_ and N_2_-treated BiVO_4_ nanoparticles were scratched off the electrode substrates and dispersed in a 0.5 M phosphate buffer solution (pH 7.2) using sonication.

### Photoelectrochemical measurements

Photoelectrochemical performances of photoanodes prepared in this work were evaluated in a typical undivided three-electrode configuration using a SP-200 potentiostat/EIS (BioLogic Science Instrument). The simulated solar illumination was obtained by passing light from a 300 W Xe arc lamp (Oriel Newport) through a water filter (IR filter)/neutral density filters/an AM 1.5G filter. Illumination through the FTO side (back-side illumination) was used. The power density of the incident light was calibrated to 100 mW cm^–2^ at the surface of the FTO substrate (before the light penetrates FTO) by using a research radiometer (International Light, IL 1700) coupled with a thermopile detector (International Light, SED623/H/N/K15) and a NREL certified reference cell (Photo Emission Tech., Inc.). The beam passed through an optical fibre and the active area of the sample exposed to electrolyte is between ca. 0.1–0.2 cm^2^. Photocurrent measurements were performed in a 0.5 M potassium phosphate (KH_2_PO_4_) buffer solution (pH 7.2) with or without 1 M sodium sulfite (Na_2_SO_3_) as a hole scavenger. Before measurements, the electrolyte was thoroughly deaerated by purging with nitrogen. Photocurrents were monitored either while sweeping the potential to the positive direction with a scan rate of 10 mV s^−1^ or while applying a constant bias. The mean values and confidence intervals of photocurrent density and electron–hole separation yield provided in the text and Supplementary Table 1 were determined using measurements on three different samples at the 95% confidence level. While all measurements were carried out using a Ag/AgCl (4 M KCl) reference electrode, all results in this work were presented against the RHE for ease of comparison with H_2_ and O_2_ redox levels and other reports that used electrolytes with different pH conditions. The conversion between potentials versus Ag/AgCl and versus RHE is determined using the equation below.


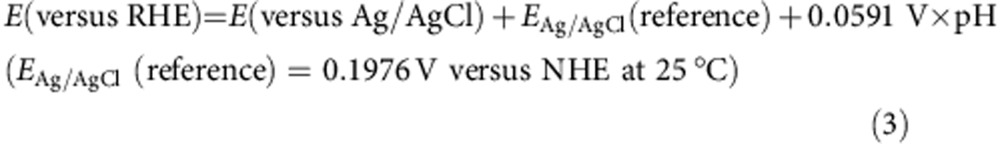


IPCE at each wavelength was determined using illumination from a 300 W Xe arc lamp (Oriel Newport) passed through an AM 1.5G filter, neutral density filters, and a water filter to approximate the output of the sun. Monochromatic light was produced using an Oriel Cornerstone 130 monochromator with a 10-nm bandpass. The intensity of incident light was measured using a research radiometer (International Light, IL 1700) with a calibrated silicon photodiode detector (International Light, SED033). IPCE was measured in 0.5 M phosphate buffer (pH 7.2) with the same three-electrode setup described above for photocurrent measurements, using a Princeton Applied Research Potentiostat/Galvanostat model 263A to apply 0.6 V versus RHE. APCE was obtained by dividing the IPCE by the light harvesting efficiency at each wavelength using the following equations (equations 4 and 5).


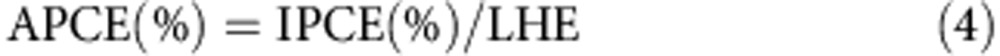



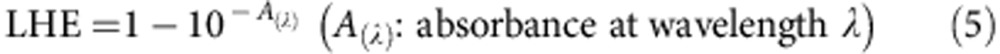


To calculate respective *J*_max_s (maximum photocurrent density achievable assuming 100% IPCE for photons with energy ≥*E*_g_) of BiVO_4_ and N_2_-treated BiVO_4_, the National Renewable Energy Laboratory (NREL) reference solar spectral irradiance at AM 1.5G (radiation energy (W m^−2^ nm^−1^) versus wavelength (nm)) was first converted to the solar energy spectrum in terms of number of photons (s^−1^ m^−2^ nm^−1^) versus wavelength (nm)^50^. Then, the number of photons above the bandgap energy of each sample shown in this study was calculated using a trapezoidal integration (in 10 nm increments) of the spectrum and was converted to the current density (mA cm^−2^). *J*_max_s for pristine BiVO_4_ and N_2_-treated BiVO_4_ were 6.47 and 9.12 mA cm^–2^, respectively.

To calculate *J*_abs_ (photocurrent assuming 100% APCE), the light harvesting efficiency at each wavelength was multiplied during each step of the trapezoidal integration. Using these calculations, *J*_abs_s for pristine BiVO_4_ and N_2_-treated BiVO_4_ were calculated to be 4.44±0.05 mA cm^–2^ and 5.30±0.07 mA cm^–2^, respectively.

Photocurrent density obtained for sulfite oxidation was used to calculate the electron–hole separation yield (=the yield of the photogenerated holes that reach the surface), *φ*_sep_, using the following equation (equation 6), where *J*_PEC_ is the measured photocurrent density and *φ*_ox_ is the yield of the surface reaching holes that are injected into the solution species^51^,^52^. For sulfite oxidation with extremely fast oxidation kinetics, surface recombination is negligible and *φ*_ox_ is ∼1. Therefore, *φ*_sep_ is obtained by dividing *J*_PEC_ by *J*_abs_.


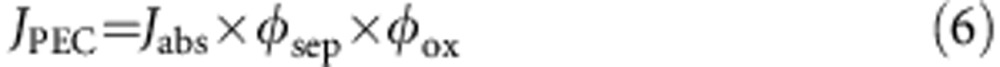


The ABPE was calculated from the *J–V* curves obtained from a two-electrode systems assuming 100% Faradaic efficiency using the following equation, where J is the photocurrent density, *V*_bias_ is the applied bias between working electrode (BiVO_4_ photoanode) and counter electrode (Pt cathode) and *P*_in_ is the incident illumination power density (AM 1.5G, 100 mW cm^–2^) (equation 7)^12^.


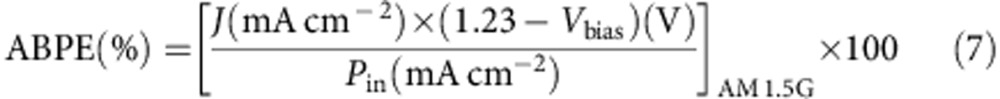


O_2_ measurements were performed using an Ocean Optics fluorescence-based oxygen sensor (Neofox, FOSPOR-R 1/16″). The probe measures the O_2_ content in the headspace as mole%, which was converted to micromoles after adjusting for the O_2_ dissolved in solution using Henry's Law. O_2_ measurements were performed with an undivided two-electrode cell using a custom built and airtight 3-neck 50 ml round-bottom flask with a N_2_-treated BiVO_4_/FeOOH/NiOOH working electrode and a Pt mesh counter electrode. O_2_ measurements were carried out while applying 0.6 V between the working electrode and the counter electrode. The experiment of oxygen evolution was run for 3 h after a 15-min waiting period at open circuit. The 15 min periods before and after the oxygen evolution step were used to calculate the O_2_ leak rate of the cell. The amount of O_2_ detected was divided by the amount of O_2_ expected calculated from photocurrent assuming 100% Faradaic efficiency to calculate the true Faradaic efficiency or the photocurrent-to-O_2_ conversion efficiency.

H_2_ production measurements were carried out by applying 0.6 V between the working electrode and the counter electrode (two-electrode system) under the same condition used in O_2_ measurement, using GC (SRI Instruments) to analyze the headspace. The amount of H_2_ gas evolved was determined by taking 100 μl of gas from the headspace of the cell using a syringe and injecting it into the gas-sampling loop of the GC every one hour. The GC was equipped with a packed MolSieve 13 X column. Helium (Airgas, ultra high purity) was used as the carrier gas. A helium ionization detector was used to quantify hydrogen concentration.

All the confidence intervals for any quantity shown in the main text were calculated using the 95% confidence level.

### DFT calculations for nitrogen substitutions

Calculations were performed with the Quantum Espresso Package^53^ using DFT with the generalized gradient approximation and the parameterization proposed by PBE^36^. We used ultrasoft pseudo-potentials, where (3*s*, 3*p*) electrons of V were included in the valence partition. A kinetic energy cutoff of 40 and 240 Ry for the wavefunction and charge density, respectively, and (4 × 3 × 6) Monkhorst–Pack k-point grids were used in geometry optimizations, and (5 × 5 × 7) k-point grids were used for density of states calculations. In our super cell calculations, both internal geometry and cell size were fully optimized for pristine BiVO_4_ and only the internal geometry was optimized for N-doped BiVO_4_.

We obtained a bandgap of 2.25 eV for pristine BiVO_4_, underestimating the experimental optical gap by only ∼0.2 eV. The good agreement between the computed bandgap and the experimental optical gap is due to a partial cancelation of errors, encompassing the PBE underestimation of gaps, the neglect of excitonic effects (the exciton binding energy is estimated to be ∼0.23 eV) and spin-orbit coupling (∼40 meV, as obtained by fully relativistic calculations). The exciton binding energy was estimated using a hydrogen-like model, 
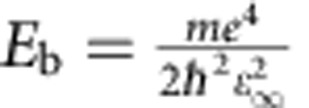
 (*E*_b_: the binding energy of the first excitonic state; m: the effective mass, 
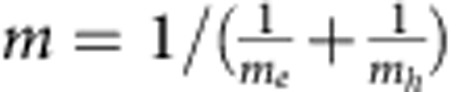
; *ɛ*_∞_: the high-frequency dielectric constant).

Our results for the band structure of the N-doped oxide (12.5% O atoms replaced with N, equivalent to the stoichiometry BiVO_3.5_N_0.5_) are shown in Supplementary Fig. 5 for the spin-polarized configuration, which was found to be energetically favoured. The 1:1 substitution of O with N cannot compensate for the charge change caused by the loss of an O atom. (Oxygen has 6 valence electrons and N has 5 valence electrons.) The resulting presence of excess holes leads to two localized empty bands (labelled ‘a' in Supplementary Fig. 5b) inside the bandgap of BiVO_4_. The energy of the localized mid-gap states is sensitive to the location of N atoms in the lattice (it varies by 0.5 eV depending on the position of the N atom). The bandgap of N-doped BiVO_4_, defined as the energy difference between the top of the valence band (level labelled ‘b' in Supplementary Fig. 5b) and the bottom of the conduction band (level labelled ‘c' in Supplementary Fig. 5b), is 1.77 eV at 12.5% N-doping, i.e. 0.48 eV lower than that of pristine BiVO_4_.

These bands are no longer present in the case of N substitution accompanied by charge-balancing O vacancies, which will serve as electron donors and compensate for the excess holes present due to N substitution (Fig. 4a). We also show the band structure of the N-doped oxide (12.5% O replaced with N) when we artificially added an electron per nitrogen to compensate for excess holes generated from charge-imbalanced N substitution (Supplementary Fig. 6). (In other words, we artificially substituted O with N^−^). Our results show that the bandgap of N doped BiVO_4_ is 1.86 eV at 12.5% N-doping, that is, 0.39 eV lower than that of pristine BiVO_4_. However, in this case the mid-gap states are no longer present. The projected density of states in Supplementary Fig. 6a shows that there is significant hybridization between N 2*p* and O 2*p* states at the top of the valence band.

### DFT calculations for oxygen vacancies

Calculations were performed using spin-polarized DFT and the PBE approximation. We also included Hubbard *U*-corrections to the *d* electrons of V (*U*=2.7 eV) following a previous study by Park *et al*.^17^. All O vacancies discussed in this study were created by removing neutral O atoms. At the PBE level of theory, when 1.5 or 6% O vacancies were introduced in the system, we observed the appearance of one isolated band inside the bandgap of BiVO_4_ (shown in Supplementary Fig. 7), whose bandgap and insulating character were however not changed. We found that the two electrons from O vacancies were ionized and localized on a V and a Bi atom, at the PBE level of theory, as shown in Supplementary Fig. 7. This makes the charge state of the O vacancy *q*=+2. Within PBE+*U*, the isolated gap states observed within PBE split into two bands, with the spin density mostly localized on V atoms for both states, as shown in Supplementary Fig. 8. The inclusion of Hubbard *U* has several effects; it leads to (i) localization of the excess electrons only on V and (ii) an increase in the energy difference between the filled gap states created by oxygen vacancies and the conduction band minimum (mainly composed of V *d* states) by 0.2 eV, compared with PBE; such differences increase with the magnitude of *U*, however the charge density remains localized on V atoms for all *U* values.

### Small polaron binding energy and mobility

The small polaron binding energy (*W*_p_) can be computed^42^ 
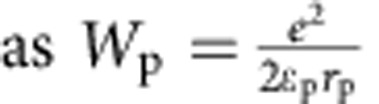
 where 
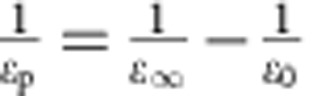
, *ɛ*_∞_ and *ɛ*_0_ are the high frequency and static dielectric constant respectively; the *r*_p_ is the small polaron radius. The small polaron radius is on the order of magnitude of 1 Å, and it can be approximately computed as 
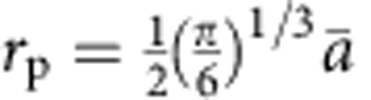
, where 
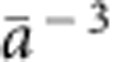
 is the number of centres per unit volume. The computed *ɛ*_∞_ and *ɛ*_0_ are 6.9 and 52, respectively, for undoped BiVO_4_ and we obtain *r*_p_=1.73 Å from our DFT calculations (at the PBE level of theory), which gives a polaron binding energy of 0.52 eV. The model adopted here yields a lower bound for the polaron binding energy. The polaron binding energy is non-negligible compared with *kT*; hence the formation of small polarons at V sites is energetically favoured.

Recent resistivity (*ρ*) measurements^39^ on single crystal n-doped BiVO_4_ show that between 250 and 400 K, the resistivity data perfectly fit to the adiabatic small polaron model^42^: 
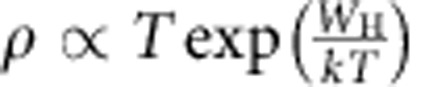
, where *T* is the absolute temperature, *k* is the Boltzmann's constant and *W*_H_ is the polaron hopping activation energy. *W*_H_ may be approximated by 
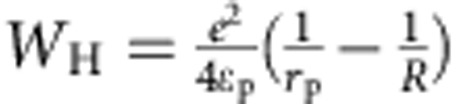
, where *ɛ*_p_ and *r*_p_ have been defined above^42^,^54^, in the formula giving the polaron binding energy. *R* is the distance between small polaron hopping centres. We found that *ɛ*_∞_ changed from 6.9 (pristine BiVO_4_) to 6.2 (doped BiVO_4_ with 6% N substitution and 3% O vacancies) and *ɛ*_0_ changed from 52 (pristine BiVO_4_) to 27 (doped BiVO_4_). The significant lowering of *ɛ*_0_ stems from the presence of several higher phonon vibrational frequencies present after N-doping (*ɛ*_0_ is inversely proportional to the square of the phonon vibrational frequencies). Overall, the *W*_H_ is lowered by 1.1% upon doping, which leads to an improvement in the mobility *μ* by ∼25% according to 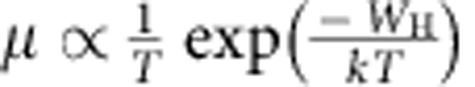.

### DFT calculations for charge-balanced N-doped BiVO_4_

Projected density of states of BiVO_4_ with charge-balanced N-doping (9% O replaced with 6% N and 3% O vacancy) obtained within DFT-PBE+*U* is shown below (Supplementary Fig. 9). In this case, due to the relative proportion of substitutional N atoms and O vacancies, electrons from O vacancies compensate for extra holes generated by N substitution, balancing the charge. For the charge-balanced N-doping, we have also carried out calculations to study the stability of an N dopant and O vacancy complex, by comparing the total energy of a configuration where the O vacancy is far away from a N dopant (at a ∼7–10 Å distance) to that of a configuration where the O vacancy is close to a N dopant (at a ∼2 Å distance) in which case N dopants and O vacancies interact strongly to form a complex; we used a super cell with two N dopants and one extra O vacancy. We found that the total energy is lowered by 0.26 eV when forming a N dopant–O vacancy complex, which means that the complex defect is thermodynamically preferred; however, the formation of this complex may be kinetically limited, as the N dopant or O vacancy have to migrate towards each other to form the complex. We note that the band structure of the sample where the N dopant–O vacancy complex is present is more dispersive than that where the N dopants and O vacancies are far apart and the bandgap is 0.2 eV higher.

### DFT calculations for N-doped BiVO_4_ with excess oxygen vacancies

We performed spin-polarized DFT with the PBE approximation calculations of BiVO_4_ with additional O vacancies, in excess to those necessary to charge N-substituted centres (doping concentration of 6% N and 6% O vacancies, see Supplementary Fig. 10). As shown in Supplementary Fig. 10, the N 2*p* states lie at the top of valence band and are mixed with O 2*p* states; the presence of N leads to an increase of the VBM energy and a decrease of the bandgap of BiVO_4_ by 0.3 eV, (The bandgap here is defined as the energy difference between the bottom of the conduction band above the Fermi level and the top of the valence band (mixed O and N 2*p* states)). In addition, there are filled gap states due to excess electrons from additional O vacancies, which are localized around V atoms, even with *U*=0. This is probably due to lattice distortions occurring in the presence of N and favouring localization of electronic states. The major findings in different doping cases are summarized in Supplementary Table 2.

## Additional information

**How to cite this article:** Kim, T. W. *et al*. Simultaneous enhancements in photon absorption and charge transport of bismuth vanadate photoanodes for solar water splitting. *Nat. Commun.* 6:8769 doi: 10.1038/ncomms9769 (2015).

## Supplementary Material

Supplementary Information Supplementary Figures 1-13 and Supplementary Tables 1-2
