# Supplementary material for: Simultaneous enhancements in photon absorption and charge transport of bismuth vanadate photoanodes for solar water splitting
Source: Nat Commun. 2015 Oct 26;6:8769. doi: 10.1038/ncomms9769 (PMC4640143; doi:10.1038/ncomms9769)
Supplement: Supplementary Information — Supplementary Figures 1-13 and Supplementary Tables 1-2 [file ncomms9769-s1.pdf]

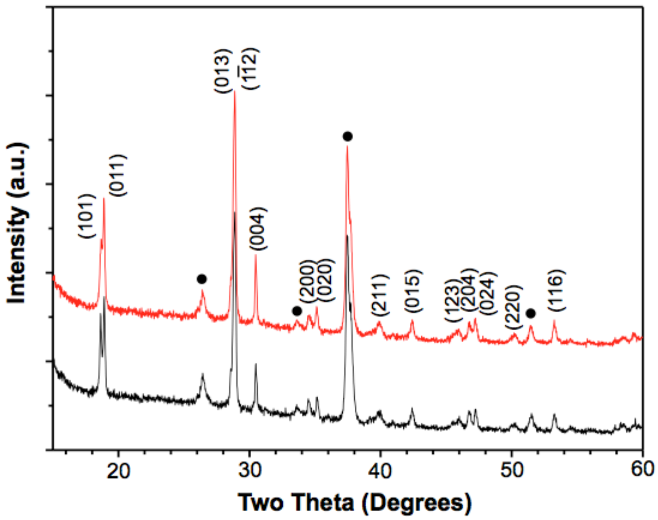

Supplementary Figure 1. XRD patterns of $BiVO_4$ (black) and N_2-treated $BiVO_4$ (red). The (hkl) indices are based on JCPDF 83-1699 (Space group: I 2/b). No detectable changes were observed by comparing these patterns probably because the peaks generated by thin nanoporous $BiVO_4$ films could not provide sufficient resolution. The peaks from FTO are indicated by black circles.

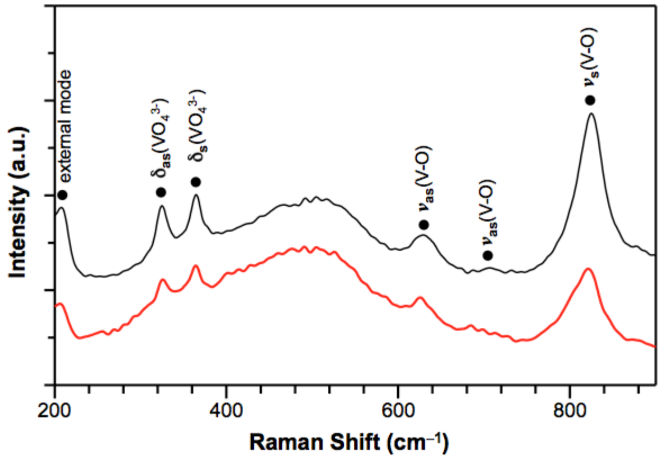

Supplementary Figure 2. Raman spectra of $BiVO_4$ (black) and N_2-treated $BiVO_4$ (red). N_2-treated $BiVO_4$ shows significant broadening of various V-O stretching and VO_4^{3-} deformation modes, suggesting that N_2 treatment indeed affected the local coordination of V in the bulk $BiVO_4$ sample (ν_s: symmetric stretching mode, ν_{as}: asymmetric stretching mode, δ_s: symmetric deformation mode, δ_{as}: asymmetric deformation mode).

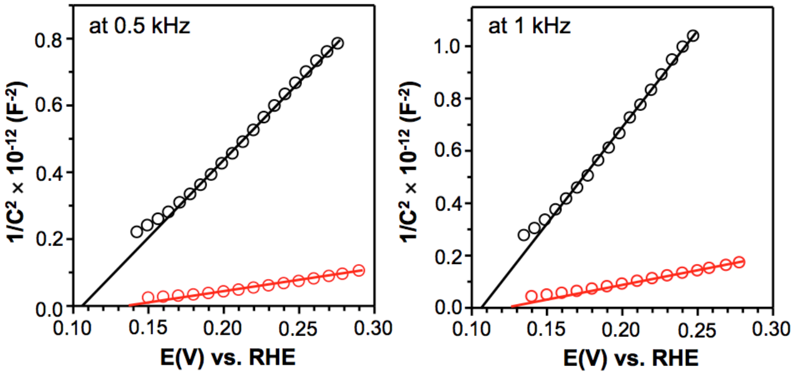

Supplementary Figure 3. Mott Schottky plots of $BiVO_4$ (black) and N_2-treated $BiVO_4$ (red) measured in a 0.5 M phosphate buffer (pH 7.2) at two different frequencies, 0.5 kHz and 1 kHz.

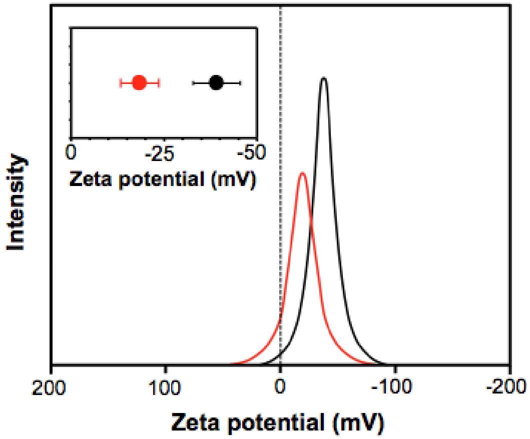

Supplementary Figure 4. Zeta potential measurement of $BiVO_4$ (black) and N_2-treated $BiVO_4$ (red) in a 0.5 M phosphate buffer solution (pH 7.2). The pH_{PZZP} of $BiVO_4$ and N_2-treated $BiVO_4$ could not be directly measured because $BiVO_4$ is not chemically stable near its $pH_{PZZP.}$ However, zeta potentials at pH 7.2 solution could be used to compare the pH_{PZZP}s of $BiVO_4$ and N_2-treated $BiVO_{4.}$ N_2-treated $BiVO_4$ has a more positive zeta potential than $BiVO_4$ at pH 7.2, which means that pH_{PZZP} of N_2-treated $BiVO_4$ is higher than that of $BiVO_4$.

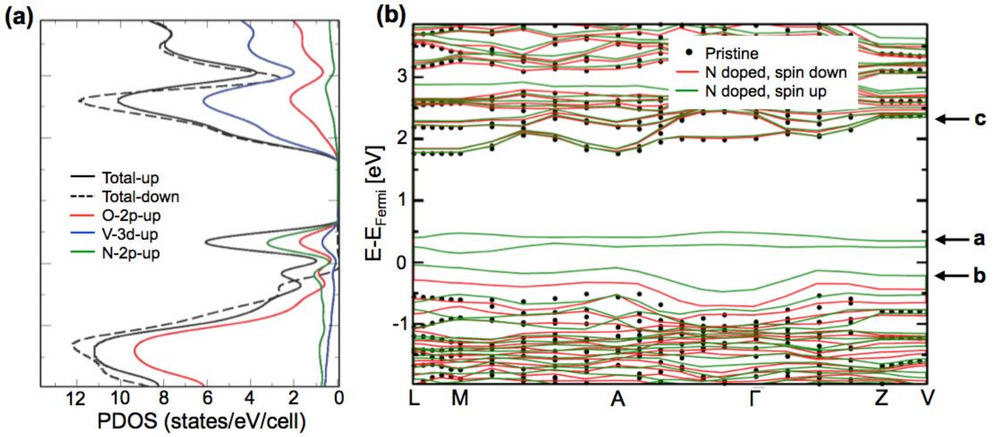

Supplementary Figure 5. (a) Projected density of states for N-doped $BiVO_4$ (12.5% O replaced with N) obtained from DFT-PBE calculations; total density of states for up (black solid) and down (black dashed) spins as well as spin up O 2p (red), V 3d (blue) and N 2p (green) states. The reference energy was set at the top of the valence band of N-doped $BiVO_4$. (b) Band structures of pristine and N-doped $BiVO_4$ (12.5% O replaced with N), obtained from DFT-PBE calculations, and aligned using the position of the 3s energy levels of V atoms that are not first neighbors of the defects.

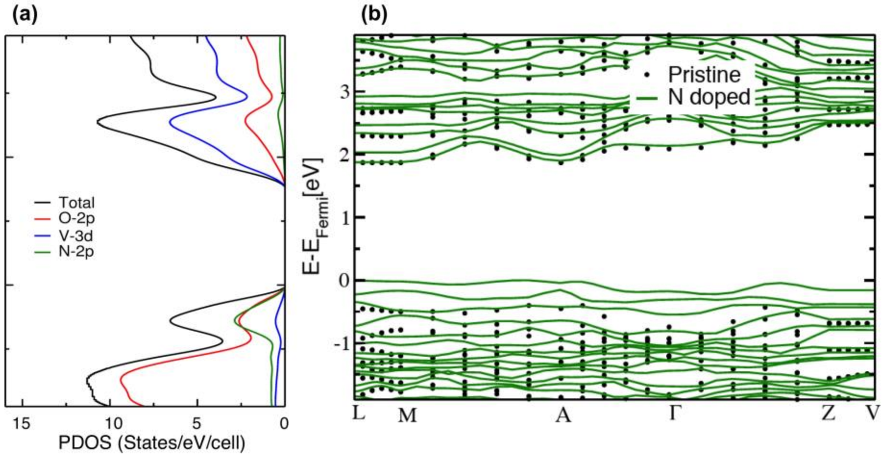

Supplementary Figure 6. (a) Projected density of states for N-doped $BiVO_4$ (12.5% O replaced with N followed by artificially adding an electron per nitrogen to compensate for the excess holes from N substitution) obtained from DFT-PBE calculations; total density of states (black solid) as well as O 2p (red), V 3d (blue) and N 2p (green) states (spin up and down states are identical). The reference energy was set at the top of the valence band of N-doped $BiVO_4$. (b) Band structures of pristine and N-doped $BiVO_4$ (12.5% O replaced with N), obtained from DFT-PBE calculations, and aligned using the position of the 3s energy levels of V atoms that are not first neighbors of the defects.

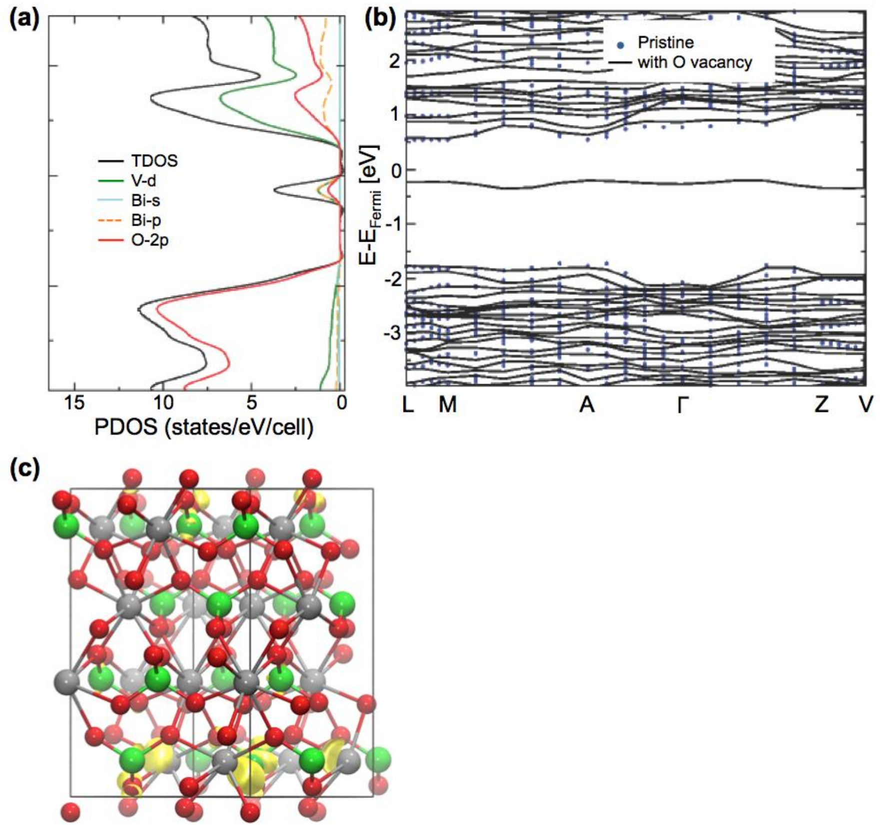

Supplementary Figure 7. (a) Projected density of states of $BiVO_4$ with 6% O vacancy obtained within DFT-PBE. The reference energy was set at the Fermi level of defective $BiVO_4$. (b) Band structures of pristine (blue dots) $BiVO_4$ and $BiVO_4$ with 6% O vacancy (black line) obtained at the PBE level of theory; the two band structures were aligned using the position of the 3s energy levels of V atoms away from the O vacancies. (c) Spin density (yellow iso-surfaces at 0.0049 e $bohr^{-3}$) of the isolated band at the Γ point; green, silver and red spheres represent V, Bi and O atoms, respectively.

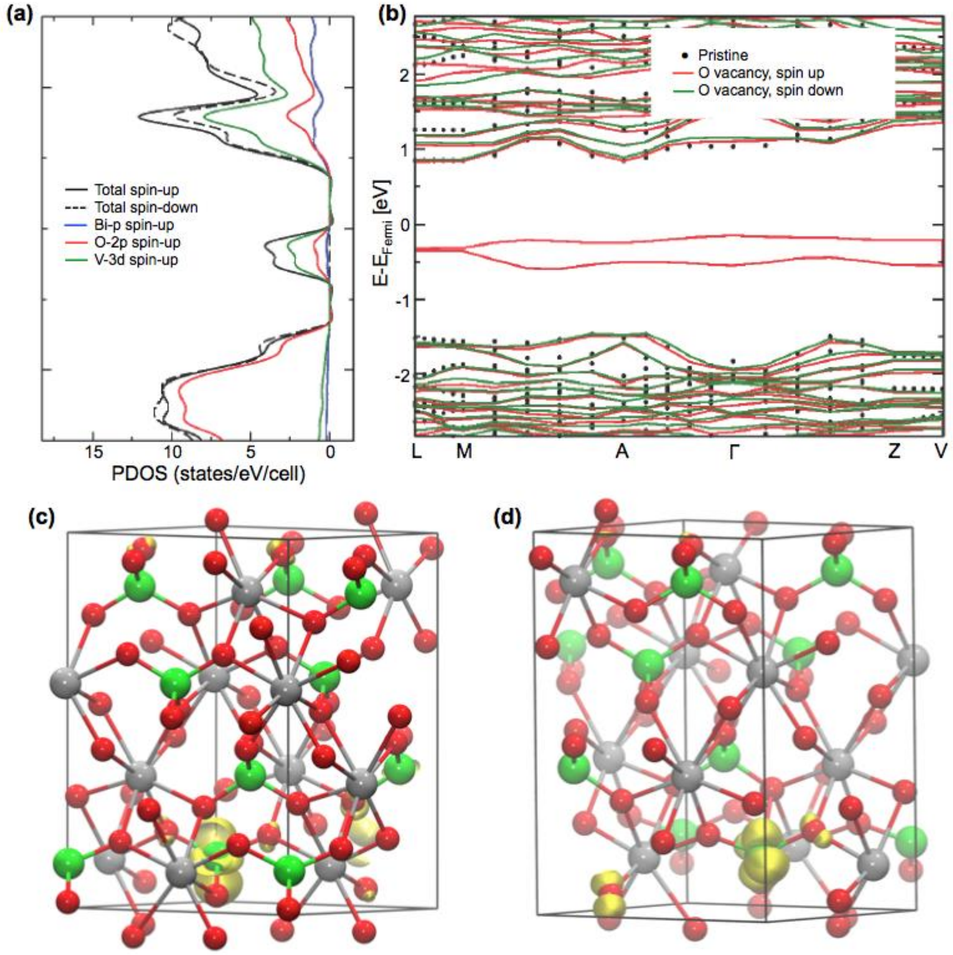

Supplementary Figure 8. (a) Projected density of states of $BiVO_4$ with 6% O vacancy obtained within DFT-PBE+U. (b) Band structures of pristine $BiVO_4$ (black dots) and $BiVO_4$ with 6% O vacancy at the PBE+U level of theory for spin up (red) and down (green). The two band structures were aligned using the position of the 3s energy levels of V atoms away from the O vacancies. The reference energy was set at the Fermi level of defective $BiVO_4$. (c) and (d) Spin density (yellow iso-surfaces at 0.0049 e $bohr^{-3}$) of the two isolated bands at the Γ point; V (green), Bi (silver), and O (red) atoms.

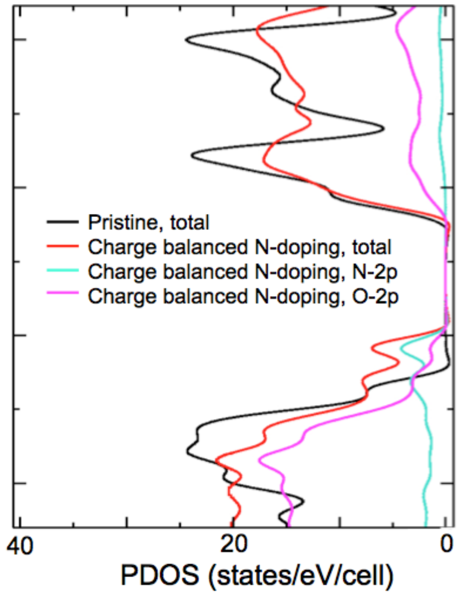

Supplementary Figure 9. Density of states of pristine $BiVO_4$ (black) and $BiVO_4$ with charge-balanced N doping (9% O replaced with 6% N and 3% O vacancy) (red) at the PBE level of theory.

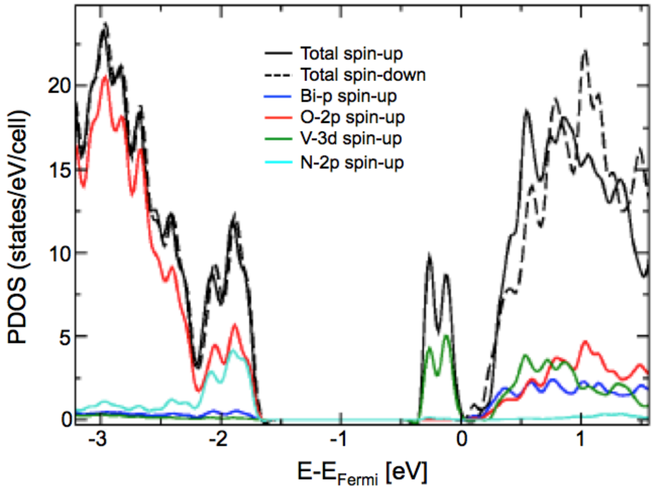

Supplementary Figure 10. (a) Projected density of states of charge-balanced N-doped $BiVO_4$ with additional O vacancies (6% N and 6% O vacancies). The reference energy is set at the Fermi level of the doped system, obtained within DFT-PBE.

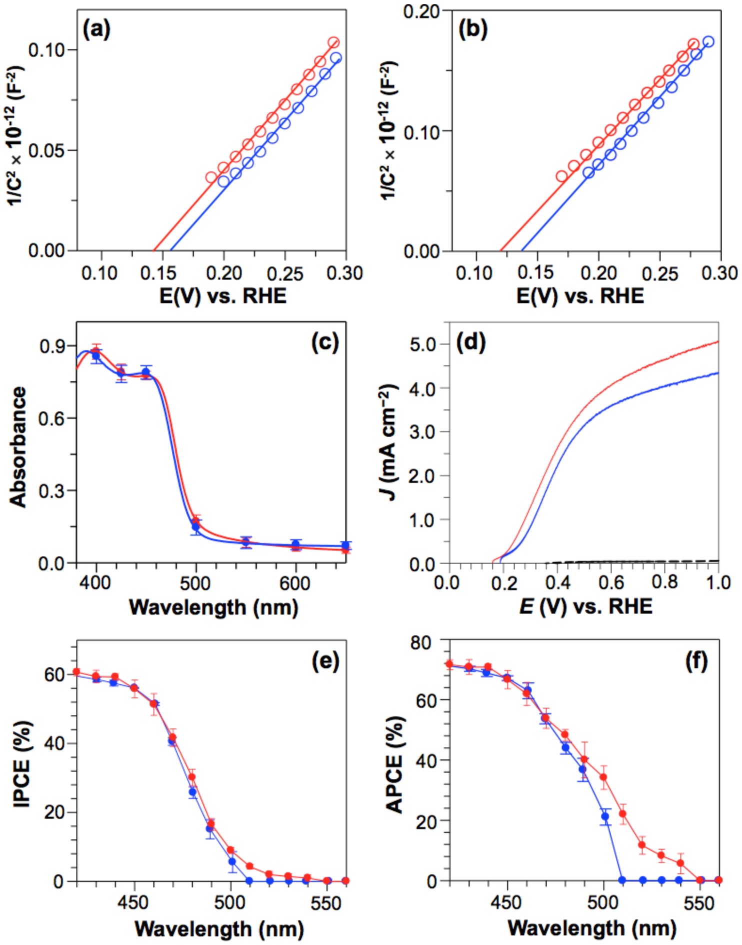

Supplementary Figure 11. Mott-Schottky plots of N_2-treated $BiVO_4$ (red) and H_2-treated $BiVO_4$ (blue) at (a) 0.5 kHz and (b) 1 kHz measured in a 0.5 M phosphate buffer (pH 7.2). (c) UV-Vis absorption spectra, (d) *J-V* plots for sulfite oxidation, (e) IPCE, and (f) APCE of N_2-treated $BiVO_4$ (red) and H_2-treated $BiVO_4$ (blue). *J-V* plots were obtained in a 0.5 M phosphate buffer (pH 7.2) containing 1 M Na_2SO_3 under AM 1.5 G, 100 mW cm^{-2} illumination (scan rate, 10 mV s^{-1}). IPCE and APCE were obtained at 0.6 V vs. RHE in the same electrolyte. H_2-treated $BiVO_4$ used in these experiments was prepared by annealing $BiVO_4$ at 350 °C for 2 h while flowing H_2, which generated oxygen vacancies in the $BiVO_4$ lattice. The comparable slopes of the Mott-Schottky plots of N_2-treated and H_2-treated samples show that they have comparable concentrations of O-vacancies serving as donors. The UV-Vis spectrum of H_2-treated $BiVO_4$ shows a significant absorption before the absorption edge (λ > 510 nm) most likely due to the presence of irregular O defects generating interband states in the bandgap region. However, IPCE and APCE plots of H_2-treated $BiVO_4$ confirm that the bandgap of H_2-treated samples is the same as untreated $BiVO_4$. The APCE comparison of N_2-treated and H_2-treated samples shows that although they have the same amount of O vacancies, the electron-hole separation of N_2-treated samples is more enhanced at 470 nm $\leq \lambda <$ 510 nm. (The photocurrent onset of H_2-treated samples is 510 nm, so APCE at $\lambda \geq$ 510 nm is not considered.) This suggests that N_2-treated samples have other factors that additionally enhance electron-hole separation. This agrees well with the calculation result that N incorporation increases the mobility of polarons and can further enhance electron transport properties and electron-hole separation. This explanation also agrees well with the results discussed in Figure S12.

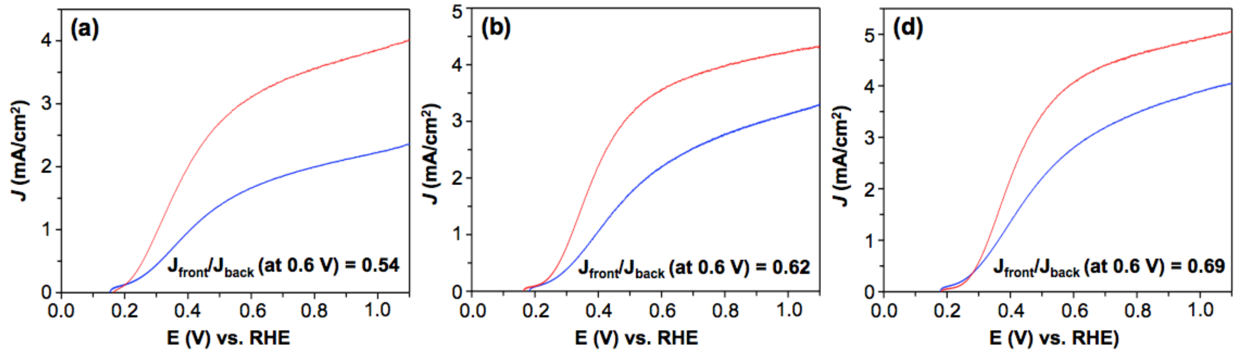

Supplementary Figure 12. Comparison of photocurrents generated from front-side illumination (blue) and back-side illumination (red) for (a) $BiVO_4$, (b) H_2-treated $BiVO_4$, and N_2-treated $BiVO_4$ in a 0.5 M phosphate buffer (pH 7.2) containing 1 M Na_2SO_3 under AM 1.5 G, 100 mW cm^{-2} illumination (scan rate, 10 mV s^{-1}). Comparing photocurrents generated from front-side illumination (J_{back}) and back-side illumination (J_{back}) of $BiVO_4$ provides information as to whether electron transport or hole transport limits the charge transport and electron-hole separation. An indication of poor electron transport being a limiting factor for electron-hole separation in $BiVO_4$ is demonstrated by $J_{front}/J_{back} < 1$ (0.54 at 0.6 V vs. RHE). The H_2-treated $BiVO_4$ shows an increase in both J_{front} and J_{back} but J_{front} increases more significantly, making J_{front}/J_{back} = 0.62. This is because O vacancies generated by H_2 treatment increase the majority carrier density and improve electron transport. The N_2-treated $BiVO_4$ also increases both J_{front} and J_{back} due to enhanced photon absorption. If N incorporation improves only the photon absorption, J_{front}/J_{back} should be similar to that of H_2-treated samples since they have similar carrier densities and, therefore, they must have similar electron transport properties. However, the N_2-treated sample shows an increase in J_{front}/J_{back} to 0.69, suggesting that N-incorporation additionally improves electron transport property via the mobility increase.

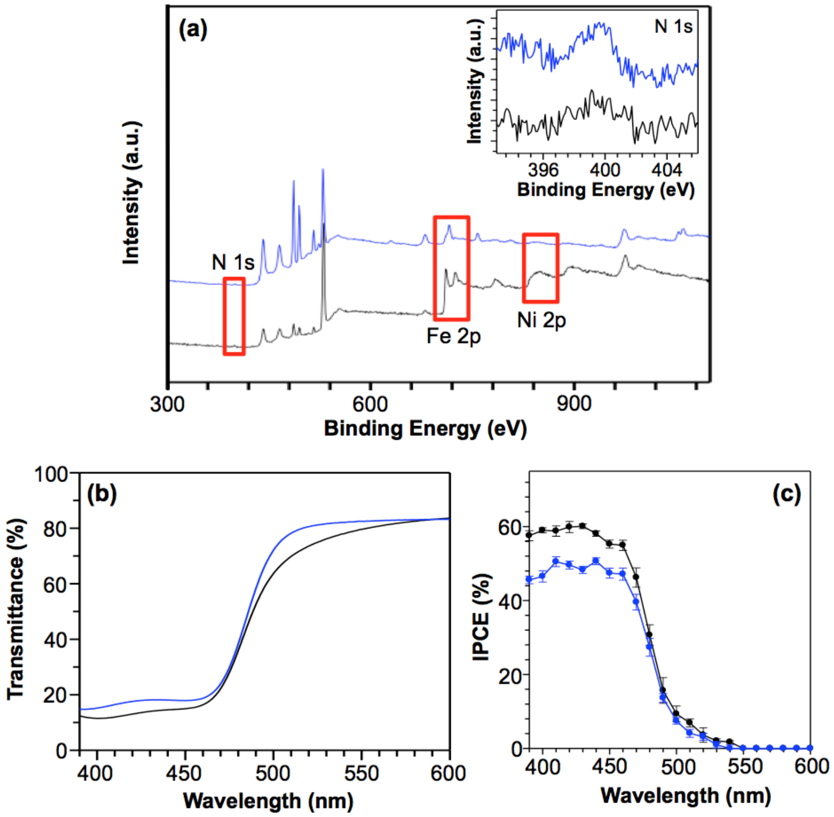

Supplementary Figure 13. (a) XPS comparison of N_2-treated $BiVO_4$/FeOOH/NiOOH before (black) and after (blue) 30 hours of photooxidation of water at 0.6 V vs. RHE in a 0.5 M phosphate buffer (pH 7.2) under AM 1.5G, 100 mW cm^{-2} illumination. The result shows a significant reduction in Ni 2p and Fe 2p peaks. The N 1s peak from N_2-treated $BiVO_4$ remained intact suggesting that N in the $BiVO_4$ is stable. (The N 1s peak after water oxidation looks slightly more enhanced due to the loss of the OEC layers covering the N_2-treated $BiVO_4$ surface.) (b) UV-Vis spectra and (c) IPCE plots of N_2-treated $BiVO_4$/FeOOH/NiOOH before (black) and after (blue) 30 hours of photooxidation of water. When the OEC coating is removed during water oxidation, the resulting accumulation of photogenerated holes at the N_2-treated $BiVO_4$ surface is expected to result in the dissolution loss of $BiVO_4$ by the photooxidation of $BiVO_4$. This is confirmed by the increase in transmittance and the decrease in IPCE in the wavelength region allowing for the bandgap transition in N_2-treated $BiVO_4$/OECs after water oxidation. However, the IPCE plot of N_2-treated $BiVO_4$/FeOOH/NiOOH still shows an earlier onset for bandgap transition confirming that N incorporated into the $BiVO_4$ lattice is stable under anodic bias and illumination. (It is more accurate to use the IPCE data than UV-Vis spectra to check the presence of N decreasing the bandgap of $BiVO_4$ because the tailing of the UV-Vis spectra below the bandgap can be very sensitive to the various defects present on the $BiVO_4$ surface and can change even when the bandgap remains the same.)

Supplementary Table 1. J_{abs}, ϕ_{sep}(at 0.6 V vs. RHE), and $J_{pec(sulfite)}$ (at 0.6 V vs. RHE) of pristine and N_2-treated $BiVO_4$. The confidence intervals were determined at the 95% confidence level using measurements on three different samples.

	J_{abs}	ϕ_{sep} (at 0.6 V vs. RHE)	$J_{pec(sulfite)}$ (at 0.6 V vs. RHE)
**Pristine**	4.44 ± 0.06 mA cm^{-2}	0.70 ± 0.04	3.27 ± 0.31 mA cm^{-2}
**N_2-treated**	5.30 ± 0.09 mA cm^{-2}	0.76 ± 0.07	4.16 ± 0.41 mA cm^{-2}

Supplementary Table 2. Summary of major findings in different doping cases.

Composition	Shift of VBM [eV]	Bandgap [eV]	Presence of mid-gap states	Polaron formation
Pristine $BiVO_4$	0.0	2.25	No	No
N-doping (12.5%)[1]	0.48	1.77	Yes	No
N-doping (12.5%) with extra electrons added to the system[2]	0.39	1.86	No	No
O vacancy (6%)[3]	0	2.25	Yes	Yes (small polarons at V)
Charge-balanced N-doping (6% N and 3% O vacancy)[4]	0.3-0.4	1.85-1.95	No	No
Charge-balanced N-doping with additional oxygen vacancies (6% N and 6% O vacancy)[5]	0.3	1.95	Yes	Yes (small polarons at V)

[1] Created by replacing 12.5% neutral O atoms with neutral N atoms.
[2] Created by replacing 12.5% neutral O atoms with neutral N atoms, followed by artificially adding one electron per nitrogen to compensate for holes generated by charge imbalanced N substitution.
[3] Created by removing 6% neutral O atoms. Two electrons at each O vacancy are spontaneously ionized and localized at V atoms. As a result, the charge state of an O vacancy is q = +2 and two small polarons are formed at V.
[4] Created by replacing 9% neutral O atoms with 6% neutral N atoms and 3% O vacancy. The electrons generated by O vacancies neutralize the holes generated by N substitution, balancing the charge.
[5] Created by replacing 9% neutral O atoms with 6% neutral N atoms and 3% O vacancy, followed by additional removal of 3% neutral O atoms. The electrons from 3% O vacancies are used to compensate for the charge change by N substitution and the electrons from the other 3% O vacancies are ionized and localized at V atoms, forming small polarons.
(1)
